# Clinical significance of substrate characteristics and ablation outcomes in patients with atrial fibrillation and significant functional mitral regurgitation

**DOI:** 10.3389/fcvm.2023.1265890

**Published:** 2023-10-25

**Authors:** Jose Antonio L. Bautista, Chin-Yu Lin, Chi-Ting Lu, Li-Wei Lo, Yenn-Jiang Lin, Shih-Lin Chang, Yu-Feng Hu, Fa-Po Chung, Ta-Chuan Tuan, Tze-Fan Chao, Jo-Nan Liao, Ting-Yung Chang, Ling Kuo, Chih-Min Liu, Shin-Huei Liu, Cheng-I Wu, Ming-Jen Kuo, Guan-Yi Li, Yu-Shan Huang, Shang-Ju Wu, Yoon Kee Siow, Ngoc Nguyen Dinh Son, Dat Cao Tran, Shih-Ann Chen

**Affiliations:** ^1^ Division of Cardiology, Department of Medicine, Heart Rhythm Center, Taipei Veterans General Hospital, Taipei City, Taiwan; ^2^ Section of Clinical Cardiac Electrophysiology, Heart Institute, St. Luke’s Medical Center – Global City, Taguig City, Philippines; ^3^ Department of Medicine, National Yang Ming Chiao Tung University, Taipei City, Taiwan; ^4^ Department of Cardiology, Taichung Veterans General Hospital, Taichung City, Taiwan; ^5^ Department of Cardiology, Serdang Hospital, Selangor, Malaysia; ^6^ Department of Medicine, University Medical Center, Ho Chi Minh City, Vietnam; ^7^ Department of Medicine, Cho Ray Hospital, Ho Chi Minh City, Vietnam; ^8^ Department of Medicine, National Chung Hsing University, Taichung City, Taiwan

**Keywords:** atrial fibrillation, mitral regurgitation, substrate, ablation, echocardiography atrial fibrillation, catheter ablation, scar

## Abstract

**Background:**

Atrial fibrillation (AF) and mitral regurgitation (MR) have a complex interplay. Catheter ablation (CA) of AF may be a potential method to improve the severity of MR in AF patients.

**Methods:**

Patients with symptomatic AF and moderate to severe MR who underwent catheter ablation from 2011 to 2021 were retrospectively included in the study. Patients' baseline characteristics and electrophysiological features were examined. These patients were classified as group 1 with improved MR and group 2 with refractory MR after CA.

**Results:**

Fifty patients (age 60.2 ± 11.6 years, 29 males) were included in the study (32 in group 1 and 18 in group 2). Group 1 patients had a lower CHA_2_DS_2_-VASc score (1.7 ± 1.5 vs. 2.7 ± 1.5, *P* = 0.005) and had a lower incidence of hypertension (28.1% vs. 66.7%, *P* = 0.007) and diabetes mellitus (3.1% vs. 22.2%, *P* = 0.031) as compared to group 2 patients. Electroanatomic three-dimensional (3D) mapping showed that group 1 patients demonstrated less scars on the posterior bottom of the left atrium compared to group 2 patients (12.5% vs. 66.7%, *P* < 0.001). AF recurrence was not different between the two groups. After multivariate logistic regression analysis, a posterior bottom scar in the left atrium independently predicted refractory MR despite successful AF ablation.

**Conclusion:**

Most patients with AF and MR showed improvement of MR after AF ablation. A scar involving the posterior bottom of the left atrium is associated with poor recovery of MR.

## Introduction

Atrial fibrillation (AF) and mitral regurgitation (MR) have a complex interplay. MR has been known to be a contributor to left atrial damage and subsequent AF if left untreated. MR may lead to AF via left atrial (LA) dilatation, while AF may also cause functional MR by dilatation of the mitral annulus in the absence of LV dilation, so-called atrial functional MR (1). In functional MR, it has been shown that such regurgitation is not an independent risk factor for predicting recurrence of catheter ablation for AF (27).

One study has shown that restoring sinus rhythm (SR) through catheter ablation (CA) of AF led to lower rates of MR along with improvement in LA and right atrium (RA) size (2). However, in some cases, there was still persistence of MR despite catheter ablation (CA) (3–5). A study by Nakamura et al. (5) was only able to identify 3 patients with left atrial scars on voltage mapping, but it could not be statistically analyzed.

Expansion of the LA wall leads to deviation of the posterior annulus toward the outside of the myocardium, causing tethering of the posterior leaflet (6–8). Silbiger et al. proposed this phenomenon of “atriogenic leaflet tethering” as a potential mechanism causing worsening MR in patients with mitral annular dilatation (7). This structural remodeling, therefore, might cause posterior wall scaring.

We hypothesize then, that the location of a scar in the left atrium may play a role in the recurrence and persistence of MR among these patients. We then wanted to explore the clinical significance of substrate characteristics and ablation outcomes in patients with AF and MR.

## Methods

### Patient selection

A total of 50 patients with moderate to severe secondary functional MR who underwent catheter ablation for AF at Taipei Veterans General Hospital (TVGH), Taipei City, Taiwan, from June 2011 to September 2021 were retrospectively evaluated. Patients with primary MR were excluded. Among them, 38 patients had moderate MR and 12 patients had severe MR. These patients were then divided into two groups: those whose MR improved to trivial or mild MR after CA of AF (group 1) and those whose MR remained moderate or severe after CA (group 2). Informed consent prior to the ablation procedure was provided to all the patients. Records of patients included in the study were then retrospectively reviewed, documenting each baseline characteristic of the patients including age, sex and co-morbidities, antiarrhythmic drugs (AAD) taken prior to ablation, and details of their ablation procedure. The LA in the three-dimensional (3D) electroanatomic map was subdivided into six pre-specified segments: roof, anterior wall, septal wall, posterior wall, posterior bottom, and lateral wall (19). The study was approved by the Institutional Review Board of Taipei Veterans General Hospital (IRB-TPEVGH no. 2021-11-015BC).

AF definitions and classifications were based on the 2020 ESC/EACTS guidelines for the diagnosis and management of AF (9). Definitions of the severity of MR were based on the recommendations by the American Society of Echocardiography (ASE) for noninvasive evaluation of native valvular regurgitation (10). Functional MR was defined as MR in patients with structurally normal valve leaflets and chordae because of an enlarged mitral annulus, leading to abnormal leaflet motion (2, 9).

### Electrophysiologic study, mapping, and ablation

Procedure methods and protocol done on included patients have been detailed in our previous studies (11–19). In brief, patients who were for electrophysiologic study and catheter ablation of AF had their antiarrhythmic medications discontinued for more than five half-lives prior to the procedure. During the procedure, a 7-French deflectable decapolar catheter with a 2-mm interelectrode distance and 5-mm spacing between each electrode pair (Abbott Inc., St. Paul, MN, United States) was placed in the coronary sinus (CS) through an access in the right internal jugular vein. Electroanatomic geometry and pre- or post-ablation voltage mapping of the LA was constructed by using one of the three available 3D electroanatomic mapping systems: CARTO 3 (Biosense Webster), EnSite NavX (St. Jude Medical), or Rhythmia (Boston Scientific).

Detailed information on high-density voltage mapping in AF has also been described by our group (17, 20). Voltage mapping of the left atrium was performed before and after ablation using multielectrode mapping catheters in the patients with paroxysmal AF. Voltage mapping was performed in the end of procedure after restoring SR in the patients with persistent AF. To ensure a detailed and complete mapping of the entire chamber, we set a density fill threshold of ≤7 and ≤5 mm for the area with bipolar voltage ≥0.5 or <0.5 respectively. Catheter contact was ascertained at the time of point collection using the tissue proximity indicator and filter available in the software on the mapping system to limit the data acquisition to within 5 mm from the original shell made with a contract force technology. Peak-to-peak global bipolar voltage of the LA were acquired at each point during sinus rhythm. Significant LVZ was defined as sites with voltage <0.50 mV.

Procedural details of CA of AF have also been detailed in our previous studies (13–15). After subsequent mapping, geometry creation and analysis, pulmonary vein (PV) isolation (PVI) was performed using an open irrigated-tip ablation catheter (ThermoCool, Biosense Webster; Flexibility, Abbott, Inc.; INTELLANAV, Boston Scientific). The said ablation catheter was used to ablate the PVs in a wide antral approach. PVI was successful after determining entrance and exit blocks of all PVs, and by achieving the absence of any electrical activity inside the PVs, or if there is presence of dissociated electrical activity within the PVs during SR. The cavotricuspid isthmus (CTI) line was performed during the procedure in patients with a history of typical atrial flutter or induced typical atrial flutter.

Other patients with paroxysmal AF also underwent pulmonary vein isolation (PVI) and non-PV trigger ablation (16). In this method, AF or atrial flutter was induced in patients with paroxysmal AF at the end of the ablation procedure. If LA flutter was induced and sustained for more than 1 min, the reentry circuit of the LA flutter was then identified by isochrone mapping, entrainment maneuvers, and post-pacing interval analysis. This was soon followed by linear ablation to eliminate atrial flutter (17).

In cases of persistent AF (PeAF) ablation, PVI was performed as described above. If AF did not stop after the first step, additional linear ablation or complex fractionated atrial electrograms (CFAEs) guided substrate ablation was performed according to the operator's discretion. If the AF became organized during ablation, electroanatomic mapping and radiofrequency ablation were performed to terminate organized tachycardia (17). If AF still persisted despite extensive ablation, external synchronized cardioversion was performed to convert AF back to SR. Non-PV trigger mapping and ablation were also performed (16).

### Post-ablation voltage mapping

A bipolar voltage map of the LA was collected shortly after completion of ablation using a multi-electrode mapping catheter. LA low voltage zones (LVZ) were identified during the mapping of the left atrium. These LA LVZ were defined by the presence of bipolar voltage amplitude of ≤0.5 mV (18). The LA was then divided into six segments (anterior wall, posterior wall, roof, posterior bottom, septal wall and lateral wall) to appropriately segregate areas where LA scars were predominant (19).

### Echocardiography

Standard two-dimensional and doppler transthoracic echocardiography (2D TTE) with color flow mapping was performed in Taipei Veterans General Hospital. Offline analysis of echocardiograms was done using a digital image management and analysis software (QLab and Xcelera multimodality cardiology image management, Philips Medical Systems, Amsterdam, Netherlands). LA anteroposterior diastolic diameters were measured using parasternal long-axis views. Left ventricular ejection fraction (LVEF) was calculated using the modified Simpson's method (20), whereby the endocardial border in both the apical four-chamber and two-chamber views in end-systole and end-diastole are traced and analyzed by the imaging software.

The determination of the MR color jet area has been mentioned previously (2). In brief, the MR color jet area was measured using apical two-chamber, apical four-chamber, and apical long-axis views. Color Doppler and the Nyquist limit were determined and set to 50–70 cm/s. Mild MR was defined as a ratio of the MR color jet area of LA area (MR/LA ratio) of ≥0.1 to <0.2, moderate MR as ≥0.2 to <0.4, and severe as ≥0.4 with an effective regurgitant orifice area (EROA) of 0.2 cm^2^ (21).

### Post-ablation follow-up and evaluation

After discharge, the patients underwent follow-up visits (2 weeks after CA, then every 1–3 months) at our cardiology clinic or with the referring physicians. Routine ECGs were obtained at each outpatient visit and a 24-h Holter electrocardiogram was performed at 3, 6, and 12 months. Review of AAD adjustments were documented at 6 months and 1 year after ablation. When the patients experienced symptoms suggestive of tachycardia after ablation, 24-h Holter monitoring or cardiac event recording was performed to define the cause of the clinical symptoms. During the follow-up, patients underwent transthoracic echocardiography at 6 months, 1 year, and more than 1 year after ablation. LVEF measurements on follow-up were determined, and the change from baseline was defined as the difference between the LVEF (△LVEF) on follow-up and baseline, divided by the baseline LVEF [(LVEF_follow-up_–LVEF_baseline_)/LVEF_baseline_]. Findings from the follow-up TTE were compared to the pre-ablation results to determine the improvement in MR.

### Statistical analyses

All statistical analyses were performed using the Statistical Package for the Social Sciences software (version 25.0, SPSS Inc., IBM Corporation, New York, USA). Descriptive statistics were used to summarize the general and clinical characteristics of included patients. Mean and standard deviations were used for continuous variables. Frequency and proportion were used for categorical values (nominal/ordinal). Differences between continuous variables were assessed using Student *t* test, whereas categorical variables were compared using the *χ*^2^ test with or without Yates correction or Fischer exact test, as indicated. A 95% confidence interval was estimated for univariate and multivariate analyses using stepwise logistic regression. Parameters with a *P* value of less than 0.1 were included in the logistic regression analysis. On the other hand, for correlations and in the multivariate analysis, a *P* value of less than 0.05 was considered statistically significant.

## Results

### Patient characteristics

The clinical characteristics of included patients are shown in Table 1. A total of fifty patients was included in the study, and then they were subdivided into those whose MR improved (group 1) and did not improve (group 2) after CA of AF. There were no significant differences in AF type between the two groups. Baseline characteristics were not significantly different between the two groups except for hypertension (group 1 vs. group 2: 28.1% vs. 66.7%, *P *= 0.007), diabetes mellitus (group 1 vs. group 2: 3.1% vs. 22.2%, *P* = 0.031), and CHA_2_DS_2_-VASc score (group 1 vs. group 2: 1.7 ± 1.0 vs. 2.7 ± 1.5, *P* = 0.005). Before ablation, there was no difference in AAD intake between groups.

**Table 1:** Baseline characteristics and echocardiographic findings of included patients.

	MR improved (group 1, *N* = 32)	MR not improved (group 2, *N* = 18)	*P*-value
Age at first ablation [in years, mean (SD)]	58.2 (±11.3)	63.7 (±10.9)	0.099
Males, *N* (%)	21 (66)	8 (44)	0.151
AF type on admission			
Paroxysmal, *N* (%)	16 (50)	9 (50)	1.0
Persistent, *N* (%)	16 (50)	9 (50)	1.0
Type of AF recurrence after ablation			
Paroxysmal, *N* (%)	10 (31.3)	9 (50)	0.197
Persistent, *N* (%)	4 (12.5)	5 (27.8)	0.184
CHA2DS2-VASc score, mean (SD)	1.7 (±1)	2.7 (±1.5)	0.005
Past medical history			
Hypertension, *N* (%)	9 (28.1)	12 (66.7)	0.007
Diabetes mellitus, *N* (%)	1 (3.1)	4 (22.2)	0.031
Coronary artery disease, *N* (%)	2 (6.2)	3 (16.7)	0.247
Prior stroke, *N* (%)	6 (18.8)	1 (5.6)	0.205
Hyperlipidemia, *N* (%)	6 (18.8)	7 (38.9)	0.124
Chronic kidney disease, *N* (%)	2 (62)	2 (11.1)	0.553
Thyroid dysfunction, *N* (%)	7 (21.9)	1 (5.6)	0.136
Anti-arrhythmic drugs taken before ablation			
Amiodarone, *N* (%)	11 (34.4)	6 (33.3)	0.942
Propafenone, *N* (%)	6 (18.8)	4 (22.2)	0.774
Dronedarone, *N* (%)	2 (6.3)	2 (11.1)	0.553
Digoxin, *N* (%)	2 (6.3)	1 (5.6)	0.923
Beta-blocker, *N* (%)	14 (43.8)	6 (33.3)	0.481
Non-DHP calcium channel blocker, *N* (%)	2 (6.3)	2 (11.1)	0.553
Flecainide	3 (9.4)	1 (5.6)	0.641
Mexiletine	2 (6.3)	0 (0)	0.228
Baseline left atrial diameter [in mm, mean (SD)]	45.2 (±8.1)	44.1 (±6.9)	0.630
Baseline left ventricular ejection fraction [in %, mean (SD)]	53.5 (±9.6)	54.6 (±11.4)	0.716
Mitral regurgitation severity on admission, *N* (%)			
Moderate	23 (71.9)	15 (83.3)	0.373
Severe	9 (28.1)	3 (116.7)	0.373

AF, atrial fibrillation; non-DHP, non-dihydropiridine; SD, standard deviation.

### Mitral valve surgery

Patients in group 1 had fewer cases of mitral valve surgery (MVS) done after CA (3.1% vs. 44.4%, *P* < 0.001). Among group 2 patients who underwent MVS after CA, one underwent mitral annuloplasty, one underwent isolated mitral valve repair, two had MitraClip procedures, and four had mitral and tricuspid valve repair.

### Ablation procedures made between groups

All patients underwent PVI during CA of AF. Patients who also underwent cavotricuspid isthmus (CTI) ablation and left atrial linear ablation procedures did not differ between groups. Non-PV trigger ablation, such as CFAE ablation, linear ablation, and SVC isolation were also similar between groups (Supplementary Table S1).

### Left atrial scar location after ablation

**Figure 1:**
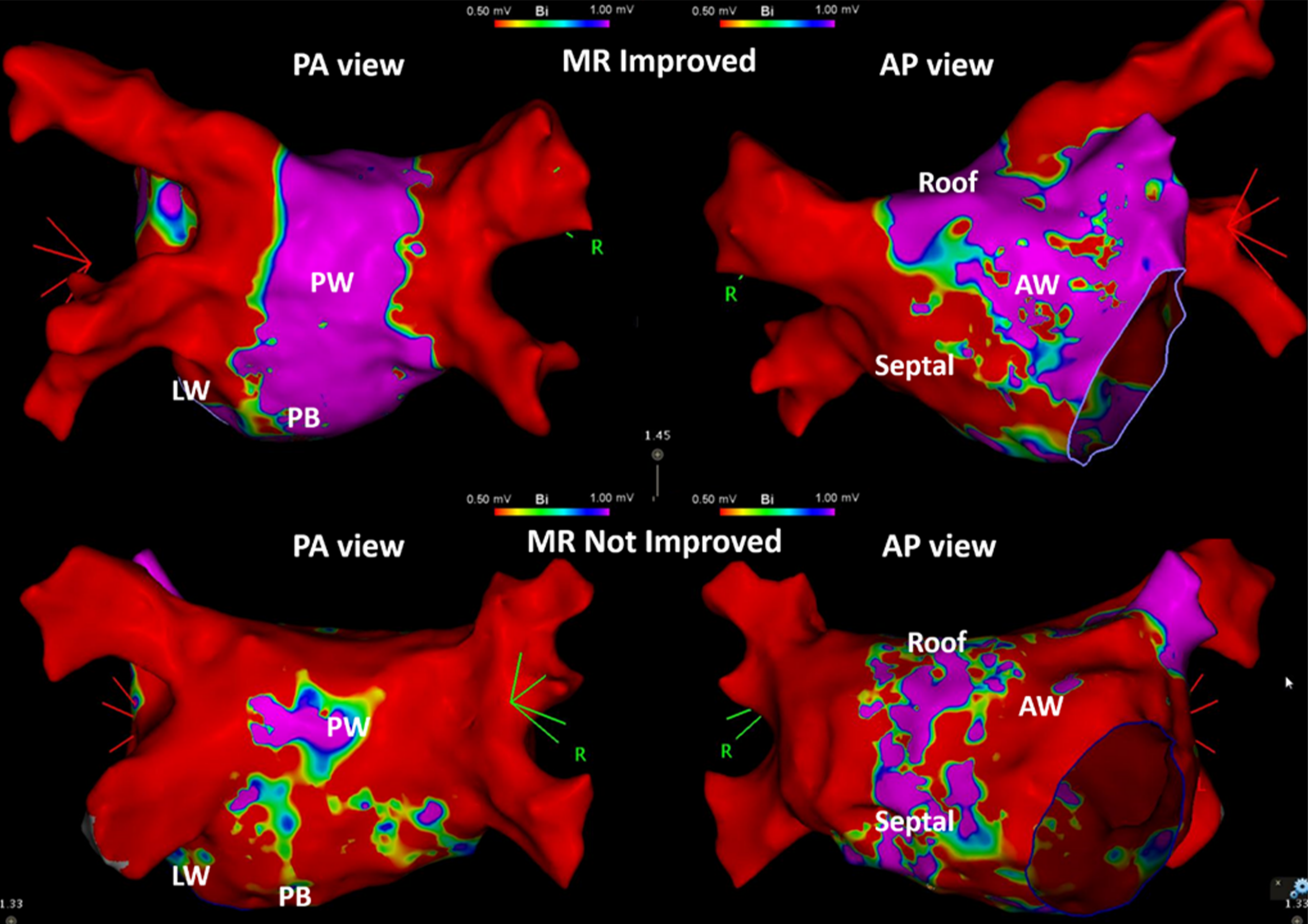
Examples of the patients in group 1 and group 2. Three-dimensional electroanatomic bipolar voltage map of the left atrium (LA) done post-ablation in patients whose mitral regurgitation (MR) responded to catheter ablation therapy (group 1, MR Improved, above) and patients whose MR did not respond to catheter ablation (group 2, MR Not Improved, below). The left-sided images show the LA in posteroanterior (PA view) projection, and the right-sided images in anteroposterior (AP view) projection. Red-colored areas indicate portions of low voltage zones (≤0.5 mV), while purple-colored areas indicate portions of voltage of more than 1 mV. Areas with blue, green, and yellow-green areas indicate voltage areas between 0.5 and 1 mV. PW, posterior wall; PB, posterior bottom; LW, lateral wall; AW, anterior wall.

**Figure 2:**
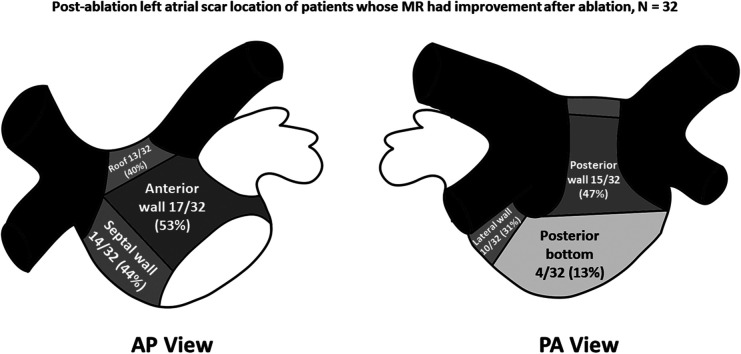
Post-ablation left atrial scar location of patients whose mitral regurgitation (MR) improved after catheter ablation of atrial fibrillation. Black-colored portions indicate pulmonary vein areas, whereas darker gray areas indicate areas of higher concentrations of low-voltage zones. Concentrations of low voltage zones are less along areas with lighter-shaded areas. AP, anteroposterior; PA, posteroanterior.

**Figure 3:**
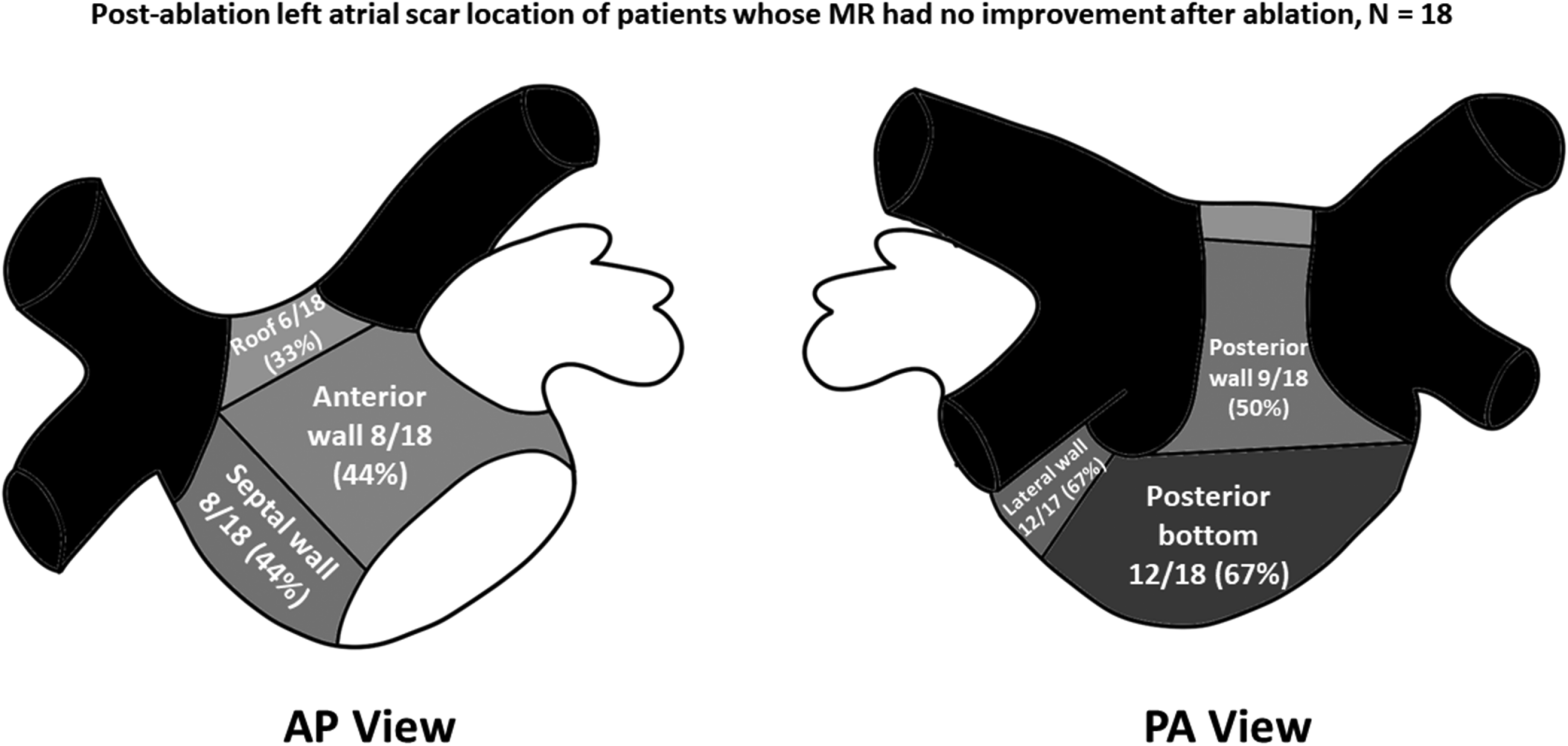
Post-ablation left atrial scar location of patients whose mitral regurgitation (MR) had no improvement after catheter ablation of atrial fibrillation. Black-colored portions indicate pulmonary vein areas, whereas darker gray areas indicate areas of higher concentrations of low-voltage zones. The concentrations of low voltage zones are less along areas with lighter-shaded areas. AP, anteroposterior; PA, posteroanterior.

Left atrial LVZ location on electroanatomic bipolar voltage mapping done post-ablation are shown on Table 2. A bipolar voltage map was acquired prior to completion of the procedure (Figure 1). LVZ locations were subdivided into 6 segments as previously mentioned (Figures 2, 3). It was shown that patients in group 1 had a less LA scar in the posterior bottom segment compared to group 2 patients (12.5% vs. 66.7%, *P* < 0.0001). LVZ locations in other segments were not significantly different between the two groups.

**Table 2:** Left atrial scar location during post-ablation electroanatomic voltage mapping.

	MR improved (*N* = 32)	MR not improved (*N* = 18)	*P*-value
Roof, *N* (%)	13 (40.6)	6 (33.3)	0.619
Anterior wall, *N* (%)	17 (53.1)	8 (44.4)	0.565
Septal wall, *N* (%)	14 (43.75)	8 (44.4)	0.963
Posterior wall, *N* (%)	15 (46.8)	9 (50)	0.836
Posterior bottom, *N* (%)	4 (12.5)	12 (66.7)	<0.0001
Lateral wall, *N* (%)	10 (31.2)	12 (66.7)	0.225

### AF recurrence after ablation

After a mean follow-up duration of 27.5 ± 22.0 months, a total of 28 patients had recurrence of AF. Recurrence of AF was significantly less among group 1 patients over group 2 patients (43.8% vs. 77.8%, *P* = 0.02). Time to recurrence of AF was not significantly different between the two groups (18.1 ± 23.6 months vs. 17.2 ± 18.6 months, *P *= 0.909). As to the type of AF after ablation, there was no difference in the number of recurrent PAF (71.4% vs. 64.3%, *P* = 0.197) and PeAF (28.6% vs. 35.7%, *P* = 0.184) between both groups.

### Anti-arrhythmic drug use after ablation

Distribution of AAD use at 6 months and 1 year after ablation are shown on Supplementary Table S2 and Supplementary Figures S1A,B. There was no difference in the use of AAD between groups after 6 months and 1 year after ablation. Among the AADs being taken after ablation, the use of beta-blockers was mostly seen on both groups (6 months, group 1 50% vs. group 2 55.6%, *P* = 0.713; 1 year group 1 41.9% vs. group 2 56.3%, *P *= 0.362).

### Mitral regurgitation severity and left ventricular ejection fraction after ablation

Left ventricular ejection fraction (LVEF) assessment on 2D TTE at 6 months and 1 year follow-up did not show any significant difference between the two groups. However, LVEF was significantly improved among group 1 patients over group 2 patients at more than 1 year follow-up (△LVEF 60 ± 5% vs. 54 ± 9%, *P *= 0.03).

### Factors predicting refractory MR after successful CA of AF

Univariate and multivariate analyses are shown on Table 3. On univariate analysis using a logistic regression model, it was shown that hypertension, a posterior bottom and lateral wall scar in the LA, baseline LVEF, and AF recurrence were predictors of refractory MR after CA of AF. However, on multivariate analysis using the same regression model, only the presence of a posterior bottom scar at the LA predicted refractory MR despite successful CA of AF.

**Table 3:** Factors associated with recurrence of atrial fibrillation among patients with mitral regurgitation.

	Univariate analysis			Multivariate analysis		
	OR	CI	*P*-value	OR	CI	*P*-value
Age	0.965	0.913–1.020	0.208			
Sex	2.386	0.732–7.780	0.149			
Hypertension	5.111	1.469–17.780	0.01	9.789	0.395–242.781	0.164
Diabetes mellitus	1.208	0.182–8.002	0.844			
Dyslipidemia	1.154	0.313–4.257	0.83			
Chronic kidney disease	1.875	0.241–14.590	0.548			
Prior stroke	0.675	0.117–3.894	0.66			
CHA2DS2-VASc score	0.682	0.428–1.084	0.106			
Cavotricuspid isthmus ablation	0.728	0.193–2.750	0.64			
CFAE	0.867	0.189–3.981	0.854			
LA linear ablation	0.636	0.197–2.058	0.45			
SVC isolation	1.875	0.241–14.590	0.548			
LA roof scar	0.731	0.218–2.444	0.611			
LA anterior wall scar	1.417	0.444–4.521	0.556			
LA septal scar	1.029	0.322–3.290	0.962			
LA posterior scar	1.607	0.502–5.141	0.424			
LA posterior bottom scar	52.500	8.574–321.457	<0.0001	109.302	3.698–3,230.223	0.007
LA lateral wall scar	3.000	0.907–9.920	0.072	0.879	0.050–15.580	0.93
Baseline left atrial diameter	1.009	0.935–1.089	0.825			
Baseline left ventricular ejection fraction	0.926	0.850–1.010	0.083	0.891	0.739–1.076	0.23
AF recurrence	4.500	1.211–16.719	0.025	24.29	0.442–1,335.511	0.119

CFAE, complex fractionated atrial electrogram; LA, left atrium; SVC, superior vena cava; AF, atrial fibrillation.

## Discussion

### Main findings

AF patients whose MR improved after CA were less hypertensive and diabetic. The MR might improve from moderate or severe MR to trivial or mild MR within 1 year after CA of AF. Patients with lesser posterior bottom scar in the LA on post-ablation bipolar voltage mapping might have improvement of MR. A significant improvement in LVEF was seen among AF patients whose MR improved after CA at more than 1 year follow-up. Also, only the presence of a posterior bottom LA scar was predictive of poor MR recovery despite successful AF ablation based on multivariate logistic regression analysis.

### Atrial fibrillation and refractory mitral regurgitation in relation to left atrial scars

Mitral regurgitation caused by AF is due to dilation of the LA from longstanding increased intra-arterial pressure, leading to mitral valve displacement, particularly the posterior mitral annulus. This displacement of the posterior mitral annulus leads to a reduction in available leaflet area for coaptation and tethering of the posterior leaflet by increasing the annual-papillary muscle distance. In the setting of AF with normal LV function, the setting of AF promotes MR by tethering of the papillary muscle to the mitral leaflets out of the annular plane (22). Also, Kagiyama and colleagues (23) identified that these patients had significantly larger anterior and posterior mitral leaflets, and a larger total leaflet area compared to patients without AF but with MR. The LV systolic dysfunction caused by a rapid and irregular heart rate in AF also leads to an impaired mitral annular systolic excursion and an imbalance in the distance between the mitral annulus and the tips of the papillary muscles (22, 25). Although previous studies have demonstrated the extent of LA remodeling on the severity of MR (25), the probability of a relationship between a more diseased LA and the persistence or recurrence of MR among AF patients after catheter ablation has not been elucidated. Based on our findings, the presence of left atrial scars, particularly scar on the posterior bottom LA, was a significant factor in causing refractory MR despite AF ablation. Scar formation in the LA is consistent with LA remodeling and is accompanied by a range of maladaptive processes that cause interstitial fibrosis of the atrial wall (27). Therefore, remodeling of the LA from extensive scars as identified by LVZs coincides with refractory MR even after AF is treated, especially if the posterior bottom LA is affected. This finding is consistent with previous studies that find LA enlargement carries important prognostic implications in patients with MR (25).

### Clinical implications

The results of the study have certain clinical implications. Early management of AF through catheter ablation can improve MR, especially if the duration of the AF is not persistent. This is consistent with findings from Gertz and colleagues wherein restoration of sinus rhythm can significantly improve atrial functional MR (2). Allowing AF to persist longer in patients has been shown to cause significant collagen deposition of the LA, particularly the posterior wall. This can lead to further scar formation within the LA, and eventually worsened outcomes in terms of arrhythmia ablation and improvement of MR. Another important implication is the control of any co-morbid conditions, particularly hypertension and diabetes. It was shown in the results of this study that patients whose MR did not improve had more cases of hypertension and diabetes. This was consistent with the findings in one study (27) that showed patients with atrial functional MR were more likely to have hypertension and diabetes. Hypertension causes left atrial wall stretching, leading to disorganization of the atrial myocardial architecture. Also, low mean LA voltage caused by scarring from a disorganized LA architecture due to hypertension can be susceptible to a stiff LA physiology (27). This pathophysiology has been shown to cause poor rhythm outcomes after AF ablation (28), and MR improvement over time. Poor MR recovery, particularly in atrial functional MR, has been independently associated with mortality over time in one study (27). Hence, it is more than emphasized, that control of comorbidities is essential to help improve AF, and most importantly, MR. Finally, catheter ablation can improve LVEF in the setting of improving MR. This improvement stems from reverse remodeling of ventricular myocardium occurring after the AF rhythm is converted back to sinus (29). This concept is supported in a study that compared the impact of catheter ablation on cardiac structural remodeling, wherein patients with MR improvement after catheter ablation of AF showed a greater increase in ejection fraction compared to those who did not have MR improvement after ablation (30).

### Comparison with previous study

Compared to previous studies that have discussed on the association and relationship between AF and MR (2–5, 31, 32), our study was able to identify a specific area within the LA endocardium, particularly the posterior bottom LA, that can predict poor recovery of MR severity after AF ablation. In one study (2) on atrial functional MR due to AF, multivariate regression showed that age, persistent AF, and mitral annular dilation were linked to MR. Consistent with their findings that atrial functional MR significantly improved with restoration of sinus rhythm, our results also showed that patients in group 1 had improvement in MR, in more than 1 year of follow-up. However, there was no analysis on the factors that were associated with poor MR recovery even after ablation. Therefore, the current study adds to the volume of information regarding atrial functional MR and AF, wherein the presence of a posterior bottom scar was predictive of poor MR recovery.

A previous study found that LA enlargement was an independent predictor of AF recurrence (30). However, our results showed that in both groups, there was no significant difference in LA size. Also, although the difference was not significant, AF recurrence was significantly less among patients whose MR improved after AF ablation, and MR severity significantly improved after a 1-year follow-up. The aforementioned study also did not analyze the association between LA enlargement and poor MR recovery. Despite our findings, it has been shown that LA enlargement on a background of AF and MR leads to more significant interstitial fibrosis of the LA myocardium with collagenous connective tissue deposition in LA posterior wall (33). Hence, it can be said that improvement of MR can be achieved as long as AF is managed early despite the size of the LA.

### Limitations

The study had several limitations. First, the number of patients in the study was small. Despite these issues, since the study focused on descriptive data, we were able to define a relatively distinct group of patients with AF who also had functional MR. No cut-off value for LVEF was provided since patients presented with AF with or without LV dysfunction. Furthermore, the study was conducted retrospectively. Therefore, prospective studies with standardized mapping catheter and uniform CA strategy are needed to prove the reproducibility of results. Another limitation was that the voltage was obtained after the ablation procedure. Such a strategy with extensive ablation might cause bias in the results. Also, cardiac magnetic resonance imaging (CMR) was not performed in this present study, and that ventricular lesions such as papillary muscle fibrosis might provide biased results (34). Hence, further study with comprehensive CMR studies before AF ablation may be done to explore this aspect. On the other hand, in cases with PeAF, we did not routinely perform cardioversion after PVI. Further prospective studies applying synchronized cardioversion right after PVI and subsequent access the LVZ might be warranted to avoid iatrogenic LVZ.

## Conclusion

Catheter ablation of AF could effectively improve MR severity within 1 year after AF ablation, especially for the patients without a scar involving the posterior bottom of the LA.

## Data Availability

The original contributions presented in the study are included in the article/**Supplementary Material**, further inquiries can be directed to the corresponding author.
