# Supplementary material for: Clinical significance of substrate characteristics and ablation outcomes in patients with atrial fibrillation and significant functional mitral regurgitation
Source: Front Cardiovasc Med. 2023 Oct 25;10:1265890. doi: 10.3389/fcvm.2023.1265890 (PMC10634397; doi:10.3389/fcvm.2023.1265890)
Supplement: Supplementary file 1 [file Table1.docx]

**Clinical significance of substrate characteristics and ablation outcomes in patients with atrial fibrillation and significant functional mitral regurgitation**

Supplementary materials

Supplementary Table 1: Procedures done between groups

Supplementary Table 2: Antiarrhythmic drug use at 6 months and 1 year after ablation

Supplementary Figure 1A: Antiarrhythmics taken by atrial fibrillation patients whose mitral regurgitation improved after catheter ablation

Supplementary Figure 1B: Antiarrhythmics taken by atrial fibrillation patients whose mitral regurgitation did not improve after catheter ablation

**Supplementary Table 1. Procedures done between groups**

|  | **MR Improved (N=32)** | **MR Not Improved (N=18)** | **P-value** |
| --- | --- | --- | --- |
| Pulmonary vein isolation, N (%) | 32 (100) | 18 (100) | N/A |
| Cavotricuspid isthmus (CTI) ablation, N (%) | 27 (84.3) | 15 (83.3) | 0.935 |
| Complex fractionated atrial electrogram (CFAE) ablation, N (%) | 5 (15.6) | 4 (22.2) | 0.569 |
| Left atrial linear ablation, N (%) | 17 (53.1) | 6 (33.3) | 0.185 |
| Superior vena cava (SVC) isolation, N (%) | 2 (6.3) | 2 (11.1) | 0.553 |

MR, mitral regurgitation

**Supplementary Table 2. Antiarrhythmic drug use at 6 months and 1 year after ablation**

|  | **MR Improved (N=32)** | **MR Not Improved (N=18)** | **P-value** |
| --- | --- | --- | --- |
| Medications taken 6 months after ablation |  |  |  |
| Amiodarone, N (%) | 8 (25.0) | 4 (22.2) | 0.83 |
| Propafenone, N (%) | 8 (25.0) | 3 (16.7) | 0.505 |
| Dronedarone, N (%) | 2 (6.3) | 1 (5.6) | 0.923 |
| Digoxin, N (%) | 0 (0) | 0 (0) | NS |
| Beta-Blocker, N (%) | 16 (50.0) | 10 (55.6) | 0.713 |
| Non-DHP Calcium Channel Blocker, N (%) | 2 (6.3) | 1 (5.6) | 0.923 |
| Flecainide | 3 (9.4) | 0 (0) | 0.188 |
| Mexiletine | 1 (3.1) | 0 (0) | 0.459 |
| Medications taken 1 year after ablation |  |  |  |
| Amiodarone, N (%) | 4 (12.9) | 5 (27.7) | 0.136 |
| Propafenone, N (%) | 7 (22.6) | 2 (11.1) | 0.416 |
| Dronedarone, N (%) | 1 (3.2) | 1 (5.6) | 0.635 |
| Digoxin, N (%) | 0 (0) | 0 (0) | NS |
| Beta-Blocker, N (%) | 13 (41.9) | 9 (50.0) | 0.362 |
| Non-DHP Calcium Channel Blocker, N (%) | 3 (9.7) | 0 (0) | 0.207 |
| Flecainide | 2 (6.5) | 0 (0) | 0.31 |
| Mexiletine | 0 (0) | 0 (0) | NS |

Non-DHP, non-dihydropiridine

**
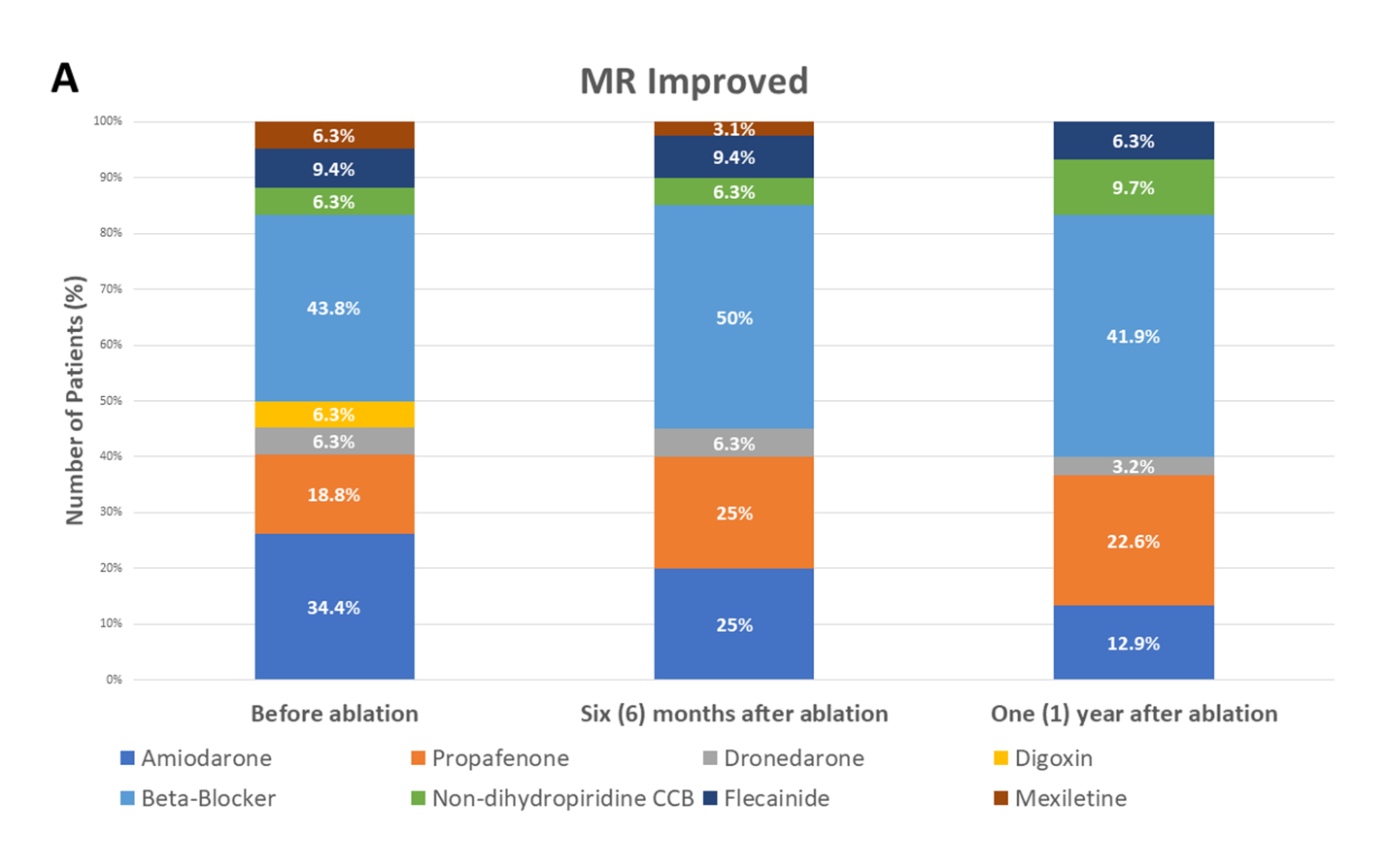
**

**Supplementary Figure 1A. Antiarrhythmics taken by atrial fibrillation patients whose mitral regurgitation improved after catheter ablation.** Bar graph showing comparison between the antiarrhythmic medications taken before, six (6) months after, and one (1) year after ablation among patients with atrial fibrillation (AF) whose mitral regurgitation (MR) improved after catheter ablation of AF. CCB, calcium channel blocker.

**
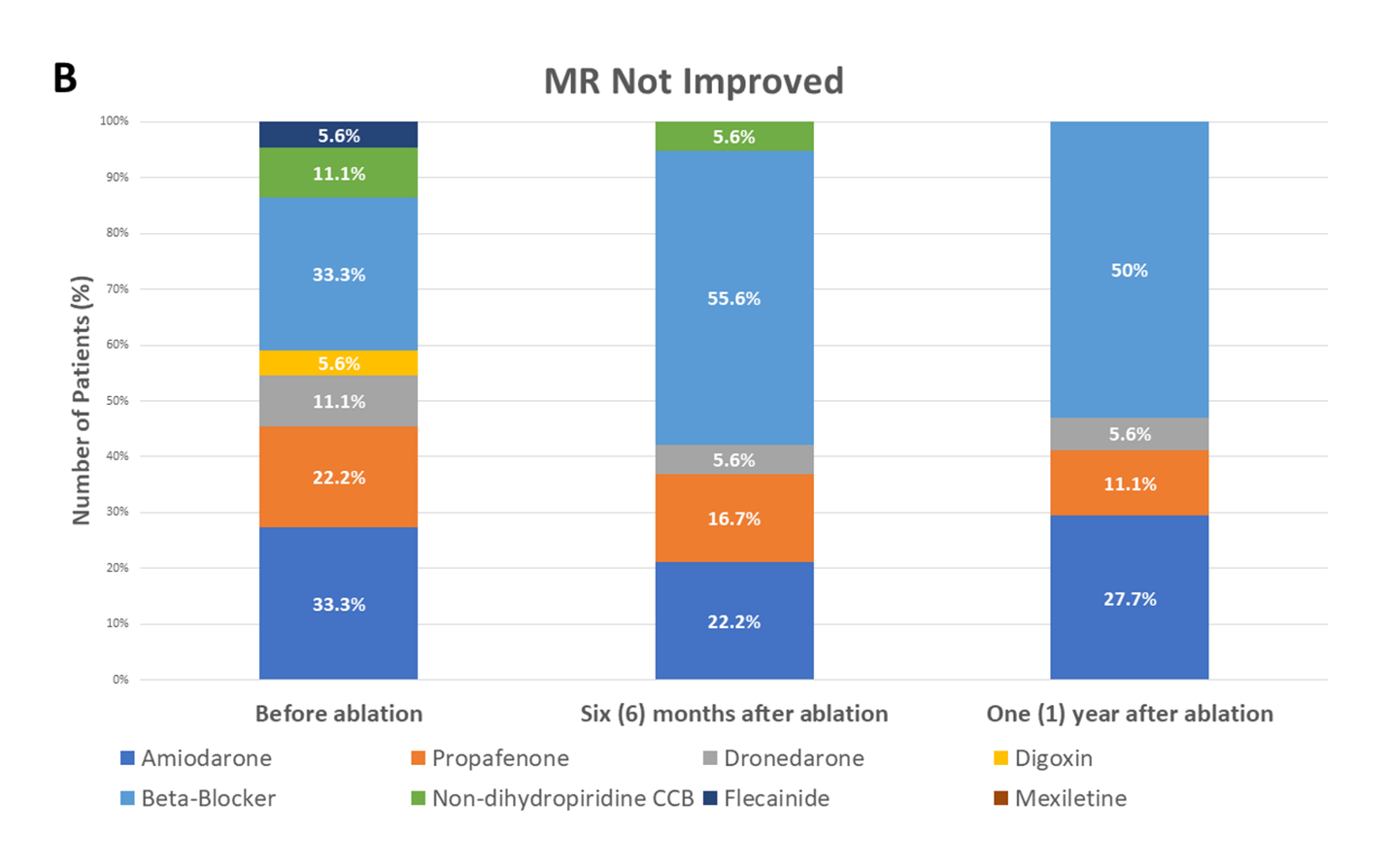
**

**Supplementary Figure 1B. Antiarrhythmics taken by atrial fibrillation patients whose mitral regurgitation did not improve after catheter ablation.** Bar graph showing comparison between the antiarrhythmic medications taken before, six (6) months after, and one (1) year after ablation among patients with atrial fibrillation (AF) whose mitral regurgitation (MR) did not improve after catheter ablation of AF. CCB, calcium channel blocker.
